# Coexistence of Rete Ovarii Cysts and Cystic Endometrial Hyperplasia in a Guinea Pig (*Cavia porcellus*)—A Detailed Clinical Case Report

**DOI:** 10.3390/vetsci13010031

**Published:** 2025-12-28

**Authors:** Mario García-González, Victoria Valiño-Cultelli, Silvia Fernández-Martín, Mónica Vila-Pastor, Antonio González-Cantalapiedra

**Affiliations:** 1 Anatomy, Animal Production and Veterinary Clinical Sciences Department, Veterinary Faculty, Universidade de Santiago de Compostela, Campus Universitario s/n, 27002 Lugo, Spain; victoria.cultelli@usc.es (V.V.-C.); silvia.fernandez@usc.es (S.F.-M.); monica.vila@usc.es (M.V.-P.); antonio.cantalapiedra@usc.es (A.G.-C.); 2 3B’s Research Group, I3B’s—Research Institute on Biomaterials, Biodegradables and Biomimetics, University of Minho, Headquarters of the European Institute of Excellence on Tissue Engineering and Regenerative Medicine, AvePark—Parque de Ciência e Tecnologia, Rua Ave 1, Edifício 1 (Sede), 4805-694 Barco, Guimarães, Portugal; 3 ICVS/3B’s—PT Government Associate Laboratory, 4806-909 Braga/Guimarães, Portugal; 4 Rof-Codina Veterinary Teaching Hospital, Faculty of Veterinary, Universidade de Santiago de Compostela, 27002 Lugo, Spain

**Keywords:** guinea pig, exotic pets, ovariohysterectomy, abdominal ultrasonography, case report, reproductive pathology

## Abstract

Reproductive system diseases are common in guinea pigs, but cases presenting with the coexistence of ovarian cysts and cystic endometrial hyperplasia (CEH) are poorly documented. This case describes a two-year-old female with abdominal tenderness and an ultrasonographic diagnosis of bilateral ovarian cysts and CEH, structured accordingly to the CARE guidelines. An ovariohysterectomy was performed via a ventral midline approach under multimodal anaesthesia and analgesia. Histopathological examination confirmed rete ovarii cysts and cystic endometrial hyperplasia. The postoperative course was uneventful, with complete recovery achieved within 21 days. To the authors’ knowledge, this is the first case report, structured accordingly to the CARE guidelines, to provide complete clinical aspects, diagnostic testing (including imaging), treatment, and final confirmation of the diagnosis of both lesions in the same patient. Abdominal ultrasonography proved to be an essential tool for the simultaneous diagnosis of ovarian and uterine abnormalities in guinea pigs. Surgical management combined with multimodal anaesthesia ensured safe outcomes and satisfactory recovery. This case report also highlights the importance and value of structuring veterinary case reports according to the CARE guidelines, thereby improving the quality, transparency, and reproducibility of clinical reporting in exotic animal medicine.

## Simple Summary

Reproductive diseases are common in domestic guinea pigs, especially in adult females. Among these disorders, ovarian cysts and uterine disease are the most frequent, although they are rarely documented in detail. This report describes a guinea pig that showed abdominal pain and was diagnosed with bilateral ovarian cysts and cystic hyperplasic change in the uterine wall. Ultrasound examination made it possible to identify both problems before surgery, allowing appropriate planning of the treatment. A surgical procedure was performed to remove the ovaries and uterus, using a safe anaesthetic protocol and a technique specifically adapted to this species. The recovery was fast and complete within three weeks. This case highlights the value of ultrasound as a diagnostic tool in small exotic animals and underlines the importance of clearly and systematically documenting clinical and surgical procedures. Doing so contributes to improving veterinary care and welfare in exotic companion species.

## 1. Introduction

Diseases of the female reproductive system are a frequent cause of morbidity in domestic guinea pigs (*Cavia porcellus*), especially in middle-aged and older females kept as companion animals. Among these, ovarian disorders, particularly cysts, are the most common and clinically significant pathology in this species [1,2,3,4]. The high incidence of ovarian cystic disease has been attributed to hormonal factors, age, reproductive status, and the absence of repeated pregnancies, since nulliparous females tend to be more predisposed [5]. In necropsies of adult guinea pigs, prevalence rates ranging from 76% to 90% have been reported [3,4].

From a pathoanatomical perspective, two principal types of ovarian cysts in guinea pigs can be distinguished: rete ovarii cysts and follicular cysts [6]. Rete ovarii cysts, of epithelial origin, are the most common and can reach several centimeters in diameter, causing compression of the adjacent ovarian parenchyma [7,8]. Follicular cysts arise from follicles that fail to ovulate but retain estrogen-secreting activity and are therefore considered functional. The pathophysiology of functional cysts closely parallels that described in cattle, being related to alterations in the hypothalamic-pituitary feedback mechanisms of the gonadal axis and the persistence of anovulatory follicles [5,6,9]. In these cases, excess estrogen can cause clinical signs including bilateral symmetrical alopecia, mammary hyperkeratosis, lethargy, and abdominal distension [4,8].

Cystic endometrial hyperplasia (CEH) is thought to develop under the combined influence of oestrogen and progesterone. Oestrogen promotes endometrial proliferation and increases progesterone receptors, while progesterone suppresses local immunity, enhances glandular secretion, and favours cervical closure, creating a uterine environment predisposed to ascending infection [10]. Consequently, CEH may coexist with endometritis or progress to a CEH–pyometra complex, a phenomenon widely documented in dogs, cats, and other domestic species [10,11,12]. In guinea pigs, although CEH is considered uncommon, its concurrence with functional ovarian cysts suggests a direct endocrine relationship [4,10,13].

The clinical diagnosis of ovarian disease requires imaging confirmation, with abdominal ultrasonography being the most sensitive and specific diagnostic tool [13]. In contrast to radiography, where the value is limited by the similar soft-tissue opacity of abdominal structures, ultrasound provides detailed information on the size, compartmentalization, and contents of the cyst, as well as the condition of the uterus and adjacent structures [4,13].

From a therapeutic standpoint, ovariohysterectomy (OVH) is the treatment of choice for ovarian cysts, especially when they are multiple, bilateral, or of considerable size [6,13]. Ultrasound-guided percutaneous drainage and the administration of GnRH agonists or hCG have been applied in selected cases; however, their results are inconsistent, and recurrences are common [8,13]. In guinea pigs presenting with concomitant uterine pathology, OVH offers the advantage of simultaneously removing the hormonal source and the affected target organ, thereby enhancing clinical recovery and reducing the risk of recurrence. The procedure may be performed via a midventral laparotomy or a unilateral flank approach, the latter being associated with reduced postoperative morbidity in younger females with a smaller uterus [10,13].

Anesthetic management in guinea pigs presents distinct challenges owing to their small body size, high surface-area-to-mass ratio, and relatively small respiratory reserve, predisposing them to hypoxia, hypoglycemia, and disturbances in thermoregulation. The use of multimodal analgesia—non-steroidal anti-inflammatory drugs (NSAIDs), opioids, and prokinetic agents—is essential, given their low pain tolerance and consequent risk of ileus [8,10].

Case reports describing the coexistence of ovarian cysts and CEH in guinea pigs are scarce in the veterinary literature [10,11], and none to date have provided combined ultrasonographic documentation of both pathologies together with a complete photographic surgical record, including wound-healing progression. In the field of exotic animal medicine, well-documented clinical cases structured according to CARE (Case Report) guidelines can play a crucial role in promoting procedural standardization and improving clinical practice [14,15]. However, no published reports concerning these concurrent diseases have explicitly adhered to these standards.

In this context, the present case report, structured according to the CARE guidelines [14,15], aims to describe an unusual case of a female guinea pig presenting with concurrent multiple ovarian cysts and uterine abnormalities, diagnosed through comprehensive abdominal ultrasonography and histopathology, and supported by clinical and photographic follow-up.

## 2. Case Presentation

For the preparation of this case report, the CARE guidelines [14,15] were followed (checklist available in the Supplementary Material File S1). The owner provided written informed consent prior to all the procedures.

### 2.1. Background

A two-year-old female guinea pig (*Cavia porcellus*) weighing 0.9 kg was presented with dysuria, which had onset two days earlier. The patient appeared lethargic but remained alert and responsive, and she continued to eat and drink normally during this period.

According to the clinical history, the animal had experienced an episode of bacterial cystitis a year previously, which had been successfully resolved with antimicrobial treatment with 25 mg/kg of oral trimethoprim-sulfamethoxazole (Septrin pediatric 8 mg/40 mg/mL, Teofarma, Pavia, Italy) every 12 h for 15 days.

The initial differential diagnosis included intestinal dysbiosis, faecal impaction, urinary tract obstruction, and the reproductive tract disease.

### 2.2. Clinical Evaluation and Diagnosis

On physical examination, the patient exhibited tachycardia (380 bpm) and tachypnoea (130 brpm), without evidence of pyrexia. Abdominal palpation elicited marked pain, evidenced by pronounced discomfort and defensive behaviour when pressure was applied to the mid-caudal abdomen. The patient showed no signs of generalised or localised alopecia. Haematological and biochemical parameters were within normal reference ranges (Table 1).

**Figure 1:**
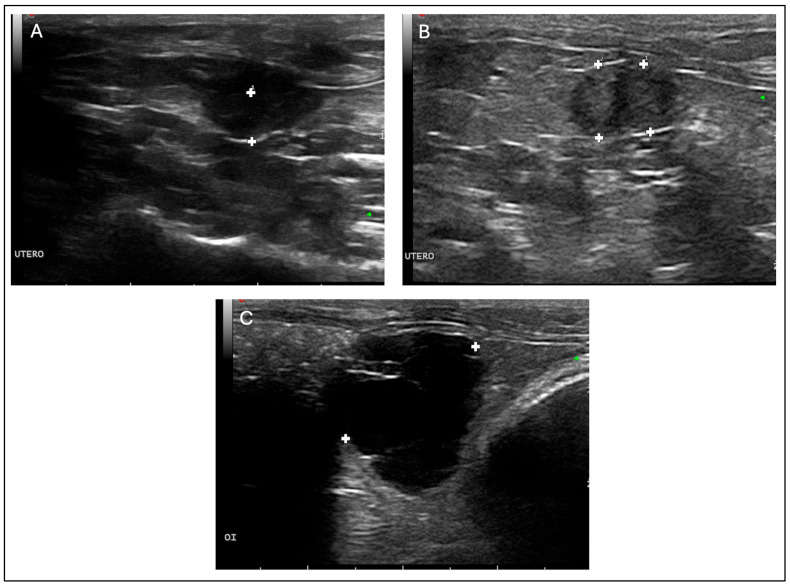
Abdominal ultrasonographic findings in a 2-year-old female guinea pig (*Cavia porcellus*). Sagittal section of the uterus body thickened (diameter: 0.37 cm) (**A**); cross-sectional view of both uterine horns thickened (left horn diameter: 0.57 cm, right horn diameter: 0.54 cm) (**B**); cross-sectional image of the left ovarian cyst (diameter: 2.3 cm) (**C**). Images were obtained using a linear probe (Esaote MyLab LA523, 12 MHz).

An abdominal ultrasound examination was performed under sedation (to minimise stress), using medetomidine (0.25 mg/Kg IM, Domtor, Esteve, Barcelona, Spain), and buprenorphine (0.03 mg/Kg IM, Buprex, RB Pharmaceuticals, Berkshire, UK) using a 12 MHz linear probe (Esaote MyLab LA523, United Medical Instruments, Inc., CA, USA). No free fluid was found. The ovaries were notably enlarged, containing multiple rounds, thin-walled anechoic cavities with smooth, well-defined contours and close apposition between adjacent cysts, showing marked posterior acoustic enhancement, consistent with clear fluid content (Figure 1C). The cystic structures lacked internal septations or suspended echogenic material, and no peripheral vascularisation was detected, suggestive of non-functional cysts of rete ovarii origin.

The uterine body and horns appeared enlarged, with thickened and irregular walls. Multiple round anechoic cavities were identified both within the uterine lumen and embedded in the endometrium. These cystic spaces had sharply delineated margins, thin walls, and homogeneously anechoic content. The uterine wall displayed a heterogeneous echotexture, characterised by increased echogenicity of the superficial endometrium and partial loss of normal stratification, findings indicative of chronic inflammatory changes or cystic endometrial hyperplasia (Figure 1A,B). No solid masses or evidence of rupture were observed. An OVH was therefore scheduled for the following day.

### 2.3. Anaesthetic Management

The patient was premedicated with a combination of ketamine (25 mg/Kg IM, Imalgène 1000, Merial, Toulouse, France), medetomidine (0.5 mg/Kg IM, Domtor, Esteve, Barcelona, Spain), and buprenorphine (0.03 mg/Kg IM, Buprex, RB Pharmaceuticals, Berkshire, UK). Meloxicam (0.2 mg/Kg SC, Metacam, Boehringer Ingelheim España, Barcelona, Spain) was administered for analgesia, and enrofloxacin (5 mg/Kg SC Ganadexil 5%, Invesa, Barcelona, Spain) was given as antibiotic prophylaxis. A 26-gauge cephalic vein catheter was placed to ensure venous access, and isotonic fluids were administered at a rate of 5 mL/kg/h throughout the procedure to maintain hydration and support circulatory stability.

Anaesthetic induction was achieved with 5% sevoflurane (Sevotek, Karizoo S.A, Barcelona, Spain) in oxygen (O_2_: 2 L/min) using a facemask, without endotracheal intubation. Anaesthesia was maintained with 2.5–3% sevoflurane (O_2_: 1.5 L/min) delivered through an Ayre’s T piece breathing circuit and mask, with spontaneous ventilation. Intraoperative monitoring parameters are summarised in Table 2. The patient was positioned in dorsal recumbency with a slight elevation of the thorax (using a small foam wedge) to facilitate diaphragmatic excursion and reduce pressure on the caudal thorax during the procedure. Thermal support was provided using an electric heating pad set to a low, controlled temperature (38.5 °C) and covered with a sterile drape.

### 2.4. Surgical Management

**Figure 2:**
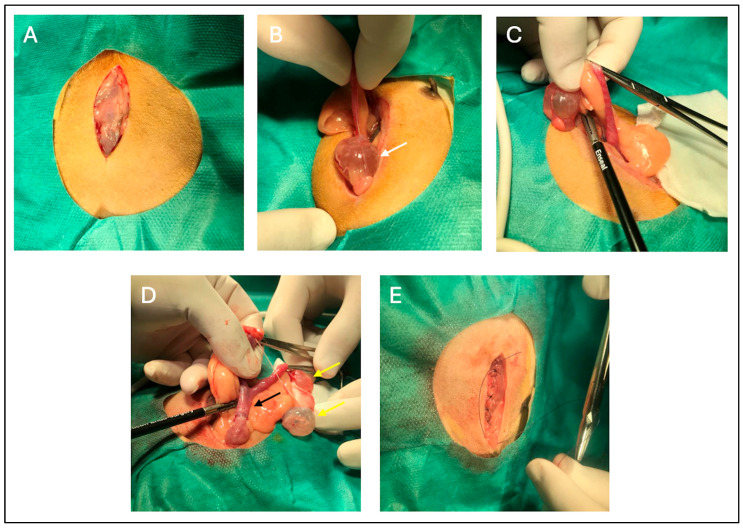
Ovariohysterectomy surgery in guinea pig (*Cavia porcellus*) with CEH and rete ovarii cysts. Midline ventral laparotomy (approximately 3 cm long) (**A**); exteriorization of the left ovary (white arrow) (**B**); dissection of the suspensory ligament and cauterization of the associated vessels (**C**); externalization of the reproductive tract (ovaries and uterus), revealing multiple thin-walled rete ovarii cysts (yellow arrows) and a congested, enlarged uterus (black arrow) (**D**); closure of the laparotomy incision (**E**).

A midline ventral laparotomy was performed following aseptic preparation of the surgical field (Figure 2A). Ovaries were identified and exteriorised, revealing multiple thin-walled cysts containing clear serous fluid (Figure 2B,C). The suspensory ligament was carefully transected, and the ovarian and mesovarian vessels were cauterised using a vessel-sealing device (Enseal^®^ model X1 curved jaw, Ethicon, Johnson & Johnson S.A., Pozuelo de Alarcon, Spain), providing precise haemostasis and minimal thermal damage.

Subsequently, the entire uterus, appearing congested and moderately enlarged, was exteriorized (Figure 2D). Surrounding adipose tissue was removed through sharp dissection, and the uterine body was transected and cauterised at the level of the cervix, with haemostasis of the ovarian and uterine stumps confirmed prior to closure, using the Enseal^®^ model X1 curved jaw device. Cecal contact was avoided by restricting traction to the reproductive tract (Figure 2B–D) and using moistened swabs to gently keep the intestinal loops away from the surgical field.

The laparotomy incision (Figure 2E) was closed in two layers. The muscular layer was sutured first, followed by the subcutaneous and intradermal layers, both using Monosyn^®^ 4-0 (B. Braun, Melsungen, Germany) in a simple continuous pattern. This intradermal closure concealed the suture line, preventing the patient from gnawing or reopening the wound. The patient tolerated anaesthesia and surgery well, with no intraoperative or postoperative adverse events.

After closing the laparotomy, the surgical wound was infiltrated with 0.5% bupivacaine (1 mg/kg SC, B. Braun, Barcelona, Spain). In the immediate postoperative period, oral meloxicam (0.2 mg/kg, Metacam, Boehringer Ingelheim España, Barcelona, Spain) was administered once daily for three days as anti-inflammatory treatment. Buprenorphine (0.01 mg/kg SC) was prescribed if additional analgesia was required. As antibiotic prophylaxis, enrofloxacin (5 mg/kg SC, Ganadexil 5%, Invesa, Barcelona, Spain) was administered once daily for three days. The wound was cleaned three times daily with sterile saline until complete healing. It was recommended to maintain sterile dressings whenever possible and to keep the wound bed clean and dry.

### 2.5. Histopathological Analysis

The excised uterine and ovarian material were submitted for routine histopathological examination.

Microscopic examination revealed dilated endometrial glands with cystic lumina, lined by simple cuboidal epithelium and supported by a moderately vascularised proliferative stroma, consistent with CEH. The ovarian parenchyma contained multiple thin-walled cystic cavities lined by flattened epithelial cells without mitotic activity, showing no inflammatory infiltrates or neoplasic changes—findings consistent with non-functional rete ovarii cysts. No evidence of purulent inflammation, necrosis, or neoplastic transformation was observed. Bacteriological culture was not performed due to financial constraints.

### 2.6. Follow-Up and Complications

The patient resumed voluntary feeding and urination within a few hours after surgery. Therefore, the use of prokinetic drugs was not necessary.

**Figure 3:**
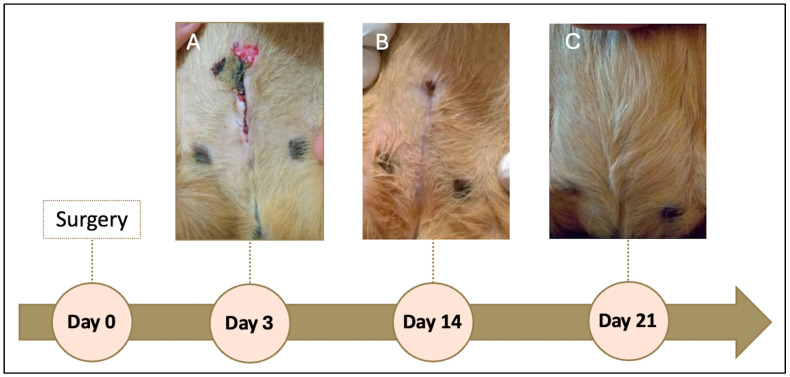
Timeline of postoperative follow-up of a guinea pig (*Cavia porcellus*) that underwent ovariohysterectomy and was diagnosed with HEC and rete ovarii cysts. Ulcerated seroma observed on day 3 (**A**); wound approximately 90% healed at 14 days after surgery (**B**); complete healing achieved by day 21, with no signs of recurrence (**C**).

On the day following the procedure, a seroma was observed, which ruptured and ulcerated on the third postoperative day (Figure 3A). The lesion was resolved with drainage, wound cleaning three times daily with sterile saline solution, and topical application of nitrofurazone (2 mg/g, Furacin, SEID Laboratories, Barcelona, Spain). Antibiotic prophylaxis was extended to 7 days to prevent secondary infection.

The wound healed without further complications. At 14 days post-surgery, approximately 90% of the incision had closed (Figure 3B), and by day 21, the wound was completely healed (Figure 3C). The patient was clinically stable, with no recurrence of discomfort or pain during urination or defecation. The prescribed buprenorphine (0.01 mg/kg SC) was not required for additional analgesia.

## 3. Discussion

This clinical case report offers a comprehensive description of the coexistence of ovarian cysts and CEH in a domestic guinea pig, integrating ultrasonographic, surgical, and histopathological findings. Beyond its clinical relevance, this case report emphasises the importance of correlating imaging with pathological anatomy and highlights the value of standardized documentation in exotic animal medicine, following structured frameworks such as the CARE guidelines [14,15].

Ovarian cysts are the most common reproductive disorder in adult female guinea pigs [1,6], and distinguishing functional (follicular) from non-functional rete ovarii cysts is clinically relevant because of their differing endocrine effects [4,7]. Functional cysts are oestrogen-secreting and may induce alopecia, behavioural changes, and uterine alterations, whereas rete ovarii cysts are non-hormonal and typically asymptomatic [6,10,17]. Ultrasonography is useful for this distinction: follicular cysts often show heterogeneous or slightly echogenic fluid, occasional internal septa, or peripheral vascularisation, reflecting their endocrine activity. In contrast, rete ovarii cysts characteristically appear as thin-walled, uniformly anechoic, avascular structures with pronounced posterior acoustic enhancement [6,10,13]. In this case, the presence of multiple smooth, anechoic, non-septated and avascular cysts strongly supported a diagnosis of non-functional rete ovarii cysts, later confirmed histologically. The coexistence of rete ovarii cysts and CEH has been reported only sporadically [10,13], and the limited number of well-illustrated cases restricts current understanding of their clinical interplay.

Ultrasonography is the diagnostic modality of choice for assessing the reproductive system of small exotic mammals, as it allows detailed evaluation of ovarian and uterine structures without the need for invasive procedures, is readily available in general practice, and avoids exposure to ionizing radiation [1,4,13]. In the present case, the ultrasound examination demonstrated the concurrent presence of multiple bilateral, thin-walled, anechoic cavities within the ovarian parenchyma, lacking septa or echogenic material, findings consistent with rete ovarii cysts. The uterine body and horns exhibited diffuse wall thickening and partial loss of mural stratification, with intramural and luminal anechoic areas compatible with CEH. The diameter of both uterine horns (0.54 and 0.57 cm) exceeded the physiological value reported by Laik-Schandelmaier et al. (2017) [7], who described an average of 3 mm in healthy females, thereby supporting the suspicion of an inflammatory or hyperplastic process. The published literature provides limited simultaneous imaging documentation of ovarian and uterine abnormalities. Most reports include only textual descriptions or macroscopic photographs, without comprehensive ultrasonographic correlation of both structures [6,10]. Even in a surgical series of 41 flank ovariohysterectomies [18], ultrasonography is mentioned as a preoperative diagnostic aid but without representative images of affected uterus and ovaries. Therefore, the present case provides comprehensive and high-quality visual documentation, contributing to the recognition of sonographic patterns compatible with rete ovarii cysts and CEH in guinea pigs.

Anesthesia in guinea pigs is particularly challenging due to their susceptibility to hypoxia, hypoglycemia, and thermoregulatory disturbances [8]. The multimodal protocol used—ketamine and medetomidine for premedication, buprenorphine and meloxicam for analgesia, and induction and sevoflurane for induction and maintenance—is supported by recent studies [10,18] and provided for a stable anesthetic plane without significant intraoperative complications. OVH remains the treatment of choice for cases involving multiple or large cysts, particularly when concurrent uterine pathology is present [1,13]. The choice of a single midline laparotomy facilitated bilateral exposure of the reproductive tract and a complete evaluation of the uterus. This approach was selected because both ovariectomy and hysterectomy were required, and the entire reproductive tract was enlarged, thereby ruling out a unilateral flank approach. The use of a vessel-sealing device (Enseal^®^ model X1 curved jaw, Ethicon, Johnson & Johnson S.A., Pozuelo de Alarcon, Spain) enabled precise hemostatic control with minimal tissue charring, reducing the risk of bleeding and promoting a smoother postoperative recovery.

The postoperative course was uneventful, with a rapid return of appetite and normal urination. The development of a seroma on the following day, which subsequently evolved into a localized abscess, is a relatively common complication after abdominal surgery and is usually associated with tissue manipulation or the presence of small subcutaneous dead spaces [19]. Its resolution through drainage, saline lavage, and topical application of nitrofurazone demonstrates that the infection was effectively controlled without the need for further intervention. The administration of subcutaneous enrofloxacin for seven days was considered appropriate as extended prophylaxis, although this must be balanced with the stress caused by repeated injections in handling-sensitive species. Compared with the oral formulation, the injectable route also minimizes the risk of dysbiosis in guinea pigs [20]. Complete healing by day 21 confirmed the satisfactory tissue repair and uncomplicated recovery.

Antibiotic selection in guinea pigs requires careful consideration due to the high risk of dysbiosis associated with many drug classes, particularly β-lactams [16]. Although culture and sensitivity testing is ideal, this is often limited in exotic pet practice by financial constraints. In this context, enrofloxacin is widely regarded as a safe empirical option in case of complications, especially injected given its low gastrointestinal impact and broad coverage [20]. In the present case, it was used only as short-term prophylaxis due to seroma suppuration in a patient with normal hematology and no clinical evidence of infection. Nevertheless, we acknowledge the increasing emphasis on responsible antimicrobial use and that culture-guided selection or non-fluoroquinolone alternatives should be considered whenever feasible (WSAVA 2018).

The prognosis for guinea pigs undergoing ovariohysterectomy due to ovarian cysts and cystic endometrial hyperplasia is generally good if surgery and anesthetic management are uneventful. In the present case, complete recovery within 21 days and the absence of recurrence confirmed an excellent long-term outcome.

Among the limitations of this report is the absence of histological photographic documentation, which was not possible due to budgetary constraints. Nevertheless, the diagnosis was supported by a formal written histopathological report confirming the presence of CEH and rete ovarii cysts. Similarly, financial limitations prevented hormonal assays (assessment of circulating hormones, particularly oestradiol and progesterone) that could have verified the presence or absence of endocrine dysfunction. Moreover, as this represents a single clinical case, the findings cannot be extrapolated in terms of incidence or therapeutic response. Finally, although hematological and biochemical analyses were within normal ranges, bacteriological culture of the uterine content was not performed, which might have provided additional insight into potential subclinical ascending infections.

The inclusion of documented clinical follow-up, high-resolution ultrasonographic images, and detailed surgical and anesthetic descriptions reinforces the value of this report as a reference for clinical practice and teaching in exotic animal medicine. Furthermore, this case was structured according to the CARE guidelines [14,15]—a feature still uncommon in veterinary publications— which enhances the transparency, reproducibility, and scientific robustness of the work, providing a useful model for future case reports.

## 4. Conclusions

The coexistence of rete ovarii cysts and CEH in guinea pigs may present with nonspecific clinical signs and requires ultrasonographic and histopathological confirmation. Ventral midline OVH, combined with a multimodal anesthetic protocol and appropriate pain management, represents a safe and effective therapeutic option, yielding favorable postoperative outcomes. This case underscores the importance of comprehensive documentation, including complete ultrasonography, surgical description, and histopathological confirmation, in the field of exotic animal medicine, where available evidence remains limited. The systematic implementation of the CARE guidelines promotes greater transparency and comparability in case reporting, thereby facilitating the development of evidence-based protocols for smaller species such as *Cavia porcellus*.

## Figures and Tables

**Table 1:** Haematological (IDEXX ProCyte Dx, IDEXX Laboratories, Barcelona, Spain) and biochemical (Catalyst Dx, IDEXX Laboratories, Barcelona, Spain) parameters obtained prior to surgery in a guinea pig (*Cavia porcellus*) with CEH and rete ovarii cysts.

**Haematological Parameter**	**Result**	Reference Range *
RBC	4.65 M/μL	3.2–8.0 M/μL
HCT	39.4%	34.0–50.0%
HGB	11.3 g/dL	10.0–17.0 g/dL
MCV	73.2 fL	71.0–96.0 fL
MCH	27.2 pg	26.0–29.0 pg
MCHC	35.0 g/dL	28.0–38.0 g/dL
RETIC	44.1 K/μL	15–150 K/μL
WBC	16.74 K/μL	5.0–18.0 K/μL
NEU	6.85 K/μL	1.10–8.60 K/μL
LYM	9.3 K/μL	1.10–12.90 K/μL
MONO	1.28 K/μL	0.10–1.80 K/μL
EOS	0.89 K/μL	0.10–1.30 K/μL
BASO	0.41 K/μL	0.00–0.50 K/μL
PLT	536 K/μL	200–700 K/μL
PDW	6.3 K/μL	6.0–12.0 fL
**Biochemical Parameter**	**Result**	**Reference Range** [16]
GLU	105 mg/dL	89.0–287.0 mg/dL
CREA	1.1 mg/dL	0.6–2.2 mg/dL
BUN	21.0 mg/dL	9.0–62.0 mg/dL
TP	5.4 g/dL	4.4–6.6 g/dL
ALB	2.9 g/dL	2.3–3.0 g/dL
GLOB	2.5 g/dL	1.7–2.6 g/dL
ALT	42.4 U/L	31.0–51.0 U/L
ALP	68.7 U/L	68.0–71.0 U/L

* Reference range according to IDEXX Laboratories.

**Table 2:** Intraoperative parameters monitored.

Parameter	Value (Mean ± SD)
Electrocardiogram	No abnormal findings
Heart rate	190 ± 10 bpm (mild bradycardia)
Respiratory rate	35 ± 5 brpm
Temperature	37.7 ± 0.1 °C
Pulse oximetry	98 ± 2%
Capnography	44 ± 2 mmHg

**bpm**: beats per minute; **brpm**: breaths per minute; **mmHg**: millimeters of mercury; **SD**: standard deviation.

## Data Availability

The original contributions presented in this study are included in the article/Supplementary Material. Further inquiries can be directed to the corresponding author(s).

## Supplementary Materials

The following supporting information can be downloaded at https://www.mdpi.com/article/10.3390/vetsci13010031/s1: Supplementary Material File S1: CARE checklist.

## Abbreviations

The following abbreviations are used in this manuscript:

bpm	Beats per minute
brpm	Breaths per minute
CEH	cystic endometrial hyperplasia
mmHg	Millimeters of mercury
NSAIDs	Non-steroidal anti-inflammatory drugs
OVH	Ovariohysterectomy
TID	Three times a day
