# Supplementary material for: Coexistence of Rete Ovarii Cysts and Cystic Endometrial Hyperplasia in a Guinea Pig (Cavia porcellus)—A Detailed Clinical Case Report
Source: Vet Sci. 2025 Dec 28;13(1):31. doi: 10.3390/vetsci13010031 (PMC12846476; doi:10.3390/vetsci13010031)
Supplement: Supplementary file 1 [file vetsci-13-00031-s001.zip › vetsci-4049007-supplementary.pdf]

CARE
case report guidelines

CARE Checklist of information to include when writing a case report

(cc) BY-NC-ND

Topic	Item	Checklist item description	Reported on Line
Title	1	The diagnosis or intervention of primary focus followed by the words "case report"	Lines 1-3
Key Words	2	2 to 5 key words that identify diagnoses or interventions in this case report, including "case report"	Lines 46-47
Abstract (no references)	3a	Introduction: What is unique about this case and what does it add to the scientific literature?	Lines 29-32
	3b	Main symptoms and/or important clinical findings	Lines 30-32
	3c	The main diagnoses, therapeutic interventions, and outcomes	Lines 30-31
	3d	Conclusion—What is the main "take-away" lesson(s) from this case?	Lines 38-44
Introduction	4	One or two paragraphs summarizing why this case is unique (**may include references**)	Lines 48-110
Patient Information	5a	De-identified patient specific information.	Line 117
	5b	Primary concerns and symptoms of the patient.	Lines 117-119
	5c	Medical, family, and psycho-social history including relevant genetic information	Lines 121-124
	5d	Relevant past interventions with outcomes	Lines 121-124
Clinical Findings	6	Describe significant physical examination (PE) and important clinical findings.	Line 129-134
Timeline	7	Historical and current information from this episode of care organized as a timeline	Figure 3
Diagnostic Assessment	8a	Diagnostic testing (such as PE, laboratory testing, imaging, surveys).	Lines 129-152; 207-216; Table 1&2
	8b	Diagnostic challenges (such as access to testing, financial, or cultural)	Lines 321-330
	8c	Diagnosis (including other diagnoses considered)	Lines 125-126
	8d	Prognosis (such as staging in oncology) where applicable	Lines 235-238
Therapeutic Intervention	9a	Types of therapeutic intervention (such as pharmacologic, surgical, preventive, self-care)	Lines 158-204; 223-238
	9b	Administration of therapeutic intervention (such as dosage, strength, duration)	Lines 158-204; 223-238
	9c	Changes in therapeutic intervention (with rationale)	Lines 226-234
Follow-up and Outcomes	10a	Clinician and patient-assessed outcomes (if available)	Lines 299-310
	10b	Important follow-up diagnostic and other test results	Lines 223-234
	10c	Intervention adherence and tolerability (How was this assessed?)	Lines 193-194
	10d	Adverse and unanticipated events	Lines 226-227
Discussion	11a	A scientific discussion of the strengths AND limitations associated with this case report	Lines 321-336
	11b	Discussion of the relevant medical literature **with references**.	Lines 248-320
	11c	The scientific rationale for any conclusions (including assessment of possible causes)	Lines 248-320
	11d	The primary "take-away" lessons of this case report (without references) in a one paragraph conclusion	Lines 338-347
Patient Perspective	12	The patient should share their perspective in one to two paragraphs on the treatment(s) they received	Not applicable
Informed Consent	13	Did the patient give informed consent? Please provide if requested	**Yes** ☒ **No** ☐
